# Supplementary material for: Investigation of the Relationship between Cardiovascular Biomarkers and Brachial–Ankle Pulse Wave Velocity in Hemodialysis Patients
Source: J Pers Med. 2022 Apr 15;12(4):636. doi: 10.3390/jpm12040636 (PMC9025475; doi:10.3390/jpm12040636)
Supplement: Supplementary file 1 [file jpm-12-00636-s001.zip › jpm-1653953-supplementary.pdf]

Journal of *Personalized Medicine*

Supplementary Table S1. List of cardiovascular biomarkers measured in this study.

Platform	Catalog No.	UniProt ID	Protein Analyte	Units
Roche Diagnostics	cobas® e 411 analyzer	P45379	High sensitivity Troponin T	ng/mL
Roche Diagnostics	cobas® e 411 analyzer	P16860	NT-proBNP	pg/mL
MILLIPLEX® map Kits	HCVD1MAG-67K	Q9NQ30	Endocan-1	pg/mL
MILLIPLEX® map Kits	HCVD1MAG-67K	Q53XY6	Placental Growth Factor	pg/mL
MILLIPLEX® map Kits	HCVD3MAG-67K	P02765	Fetuin-A	ng/mL
MILLIPLEX® map Kits	HCCBP3MAG-58K	P17931	Galectin-3	ng/mL
MILLIPLEX® map Kits	HCCBP3MAG-58K	P0733	Cathepsin D	ng/mL

Abbreviation: NT-proBNP, N-terminal pro-brain natriuretic peptide

Journal of Personalized Medicine

MDPI

Supplementary Table S2. The relationship between log-transformed brachial-ankle pulse wave velocity and clinical parameters and biochemical blood profiles in the simple linear regression model.

Characteristics	β (95%CI)	*p*-value
Age (y old)	0.01 (0.003, 0.01)	<0.01
Men (%)	0.1 (−0.01, 0.1)	0.1
Smoking history (%)	0.1 (−0.02, 0.1)	0.1
Body mass index (Kg/m²)	−0.01 (−0.02, 0.01)	0.4
Systolic blood pressure (mmHg)	0.004 (0.003, 0.01)	<0.01
Diastolic blood pressure (mmHg)	0.01 (0.003, 0.01)	<0.01
Hemodialysis vintage (mo)	−0.001 (−0.001, −0.0001)	0.03
Arteriovenous shunt		
Arteriovenous fistula	−0.1 (−0.2, 0.1)	0.2
Arteriovenous graft	Reference group	
Cause of end-stage kidney disease		
Hypertension	−0.1 (−0.2, 0.1)	0.3
Diabetes Mellitus	0.2 (0.1, 0.2)	<0.01
Glomerulonephritis	−0.1 (−0.2, -0.03)	<0.01
Others*	Reference group	
Comorbidities		
Diabetes mellitus (%)	0.2 (0.1, 0.2)	<0.01
Hypertension (%)	0.1 (0.04, 0.2)	<0.01
Hyperlipidemia (%)	0.1 (−0.03, 0.1)	0.2
Coronary artery disease (%)	0.1 (−0.02, 0.2)	0.1
Cerebrovascular disease (%)	0.1 (−0.1, 0.2)	0.3
Clinical laboratory data		
Hemoglobin (mg/dL)	0.01 (−0.02, 0.03)	0.8
Albumin (mg/dL)	−0.2 (−0.3, −0.1)	<0.01
Low density lipoprotein cholesterol (mg/dL)	−0.001 (−0.002, 0.0001)	0.2
Ion Calcium (mg/dL)	−0.1 (−0.2, −0.02)	0.02
Phosphate (mg/dL)	−0.02 (−0.1, 0.02)	0.4
C-reactive protein (mg/L)	0.1 (0.02, 0.1)	<0.01
Dialysis dose, Single pool Kt/V	−0.03 (−0.2, 0.1)	0.7

Abbreviation: Kg, kilogram; mmHg, millimeter(s) of mercury; mg, milligram; dL, deciliter; L, liter; Kt/V, dialyzer clearance of urea × dialysis time/volume of distribution of urea; ng, nanogram; mL, milliliter; pg, picogram.

*Other causes of end-stage renal disease include polycystic kidney disease, tumor, systemic lupus erythematosus, gout, interstitial nephritis

Journal of Personalized Medicine

Supplementary Table S3. Association between circulating CV biomarker levels (log-transformed) and brachial-ankle pulse wave velocity (log-transformed) in hemodialysis participants using the multivariable-adjusted linear regression model with stepwise procedure selected covariates.

Cardiovascular markers	Adjusted models*	*p*-value
hsTnT	0.1 (0.1, 0.2)	<0.01
NT-proBNP	0.01 (−0.02, 0.04)	0.4
Galectin-3	0.1 (−0.02, 0.2)	0.1
Cathepsin D	0.1 (0.01, 0.1)	0.01

Abbreviation: hsTnT, high sensitivity troponin T; NT-proBNP, N-terminal pro-brain natriuretic peptide. * Multivariable linear model demonstrated as a β with 95% CIs. The multivariable linear regression is adjusted for stepwise procedure selected covariates in the final model as follows: hsTnT covariates selection is age, diastolic blood pressure, and blood level of ion Calcium; NT-proBNP covariates selection is age, diastolic blood pressure, diabetes mellitus, and blood level of low-density lipoprotein and C-reactive protein; Galectin 3 covariates selection is age, body mass index, systolic blood pressure, diabetes mellitus, and blood level of ion calcium and C-reactive protein; Cathepsin D covariates selection is age, body mass index, systolic blood pressure, diabetes mellitus, and blood level of ion calcium and C-reactive protein.
